# Supplementary material for: CD69 prevents PLZFhi innate precursors from prematurely exiting the thymus and aborting NKT2 cell differentiation
Source: Nat Commun. 2018 Sep 14;9:3749. doi: 10.1038/s41467-018-06283-1 (PMC6138739; doi:10.1038/s41467-018-06283-1)
Supplement: Supplementary file 1 — Supplementary Information [file 41467_2018_6283_MOESM1_ESM.pdf]

CD69 prevents PLZF^{hi} innate precursors from prematurely exiting the thymus and aborting NKT2 cell differentiation

Kimura et al.

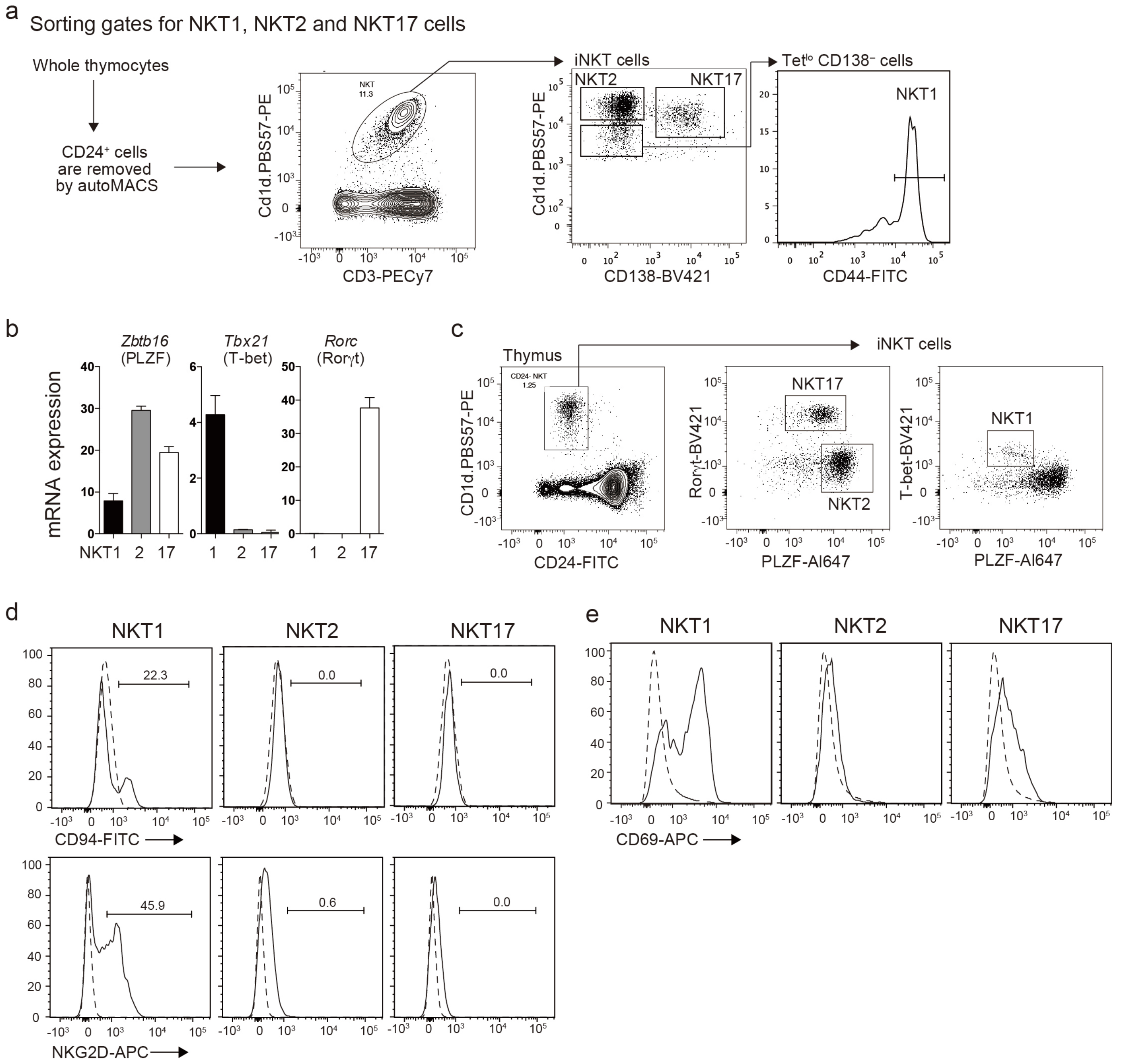

Supplementary Fig. 1: Identification of each NKT subset. (a) Sorting strategy for NKT1, NKT2 and NKT17 cells from the thymus (Fig. 1a). $CD24^+$ cells were first removed from whole thymocytes by an autoMACS cell sorter and then each iNKT subset was sorted as follows: NKT1 cells are $CD3^+$ $CD1d.PBS57^{low}$ $CD138^-$ $CD44^{hi}$, NKT2 cells are $CD3^+CD1d.PBS57^{hi}CD138^-$, and NKT17 cells are $CD3^+$ $CD1d.PBS57^+$ $CD138^+$ cells. (b) The mRNA expression of *Zbtb16*, *Tbx21* and *Rorc* relative to *Rpl13a* in NKT1, NKT2 and NKT17 cells from BALB/c-background mice prepared by cell sorting as in Fig. S1a. The mean and S.E.M. are shown. (c) Profiles of iNKT subsets in the thymus from BALB/c-background mice. NKT1, NKT2 and NKT17 subsets were identified using intracellular staining for PLZF, Rorγt and T-bet after gating on $CD24^-CD3^+CD1d/PBS\text{-}57^+$ iNKT cells. (d, e) Histograms of the CD94, NKG2D and CD69 expression on NKT1, NKT2 and NKT17 cells in the thymus from BALB/c-background mice. The dashed line indicates the isotype control. Representative data from more than 10 experiments with more than 10 mice (a, c), 5 experiments with 5 mice (b), 2 experiments with 6 mice (d) and 3 experiments with 4 mice (e) are shown.

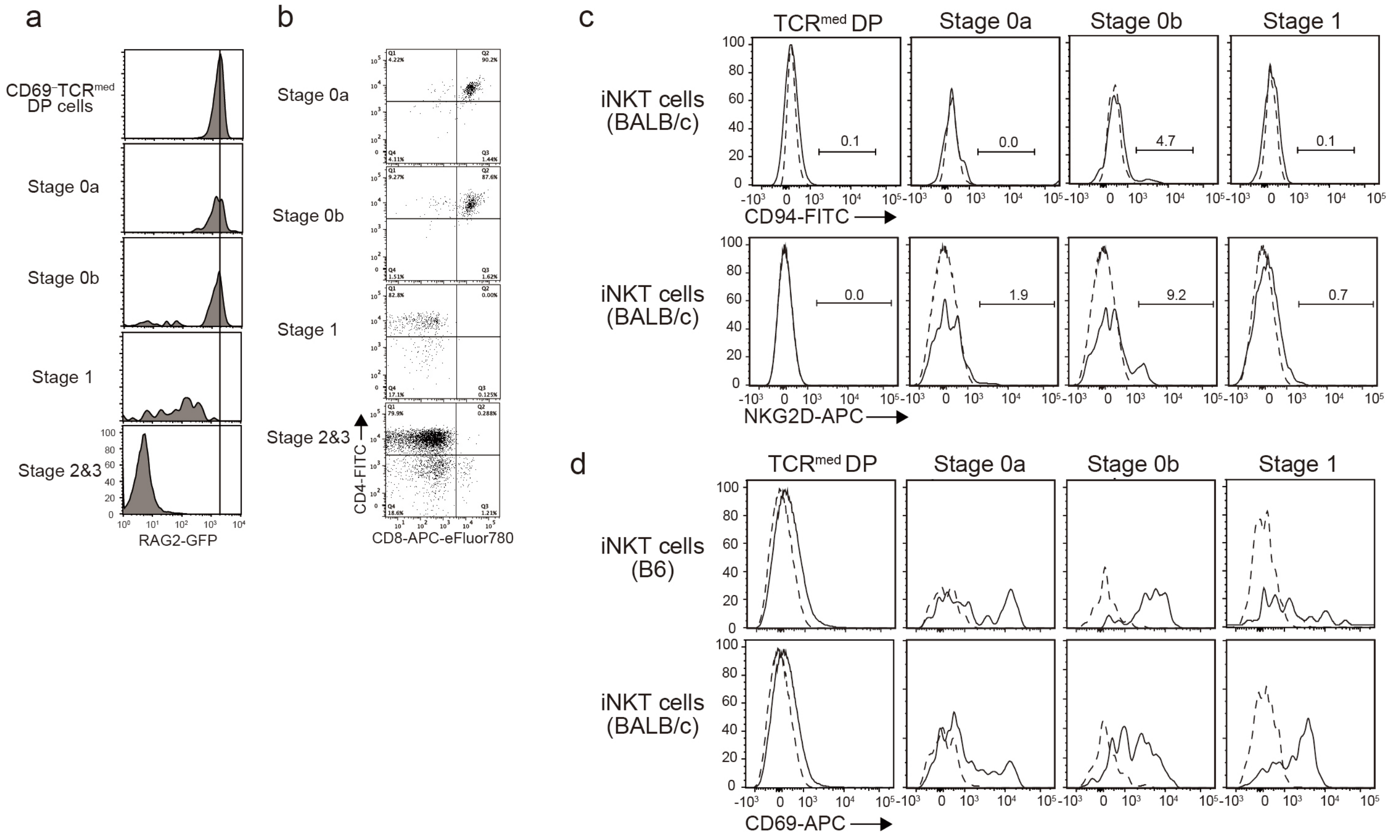

Supplementary Fig. 2: Phenotype of each stage of iNKT subsets. (a) The Rag2-GFP expression of gated $CD69^{-}TCR^{lo\text{-}med}$ DP cells, stage 0a ($CD24^{+}CD44^{low}$), stage 0b ($CD24^{+}CD44^{hi}$), stage 1 ($CD24^{-}CD44^{low}$) and stage 2 and 3 cells ($CD24^{-}CD44^{low}$) of $CD3^{+}$CD1d.$PBS57^{+}$ cells from the thymus of *RAG2*^{GFP} mice. (b) CD4/CD8 profiles of gated stage 0a ($CD24^{+}CD44^{low}$), stage 0b ($CD24^{+}CD44^{hi}$), stage 1 ($CD24^{-}CD44^{low}$) and stage 2 and 3 cells ($CD24^{-}CD44^{low}$) of $CD3^{+}$CD1d.$PBS57^{+}$ cells from BALB/c thymus. (c) The CD94 and NKG2D expression on the cells in the thymus from BALB/c-background mice, gated as in Fig. 2a and Supplementary Fig. 2a. The dashed line indicates the isotype control. (d) The CD69 expression on the cells in the thymus from either B6 or BALB/c-background mice, gated as in Fig. 2a and Supplementary Fig. 2a. The dashed line indicates the isotype control. Representative data from 4 experiments with 7 mice (a), 2 experiments with 3 mice (b), 2 experiments with 6 mice (c), and 3 experiments with 3 (C57BL/6) or 6 mice (BALB/c) (d) are shown.

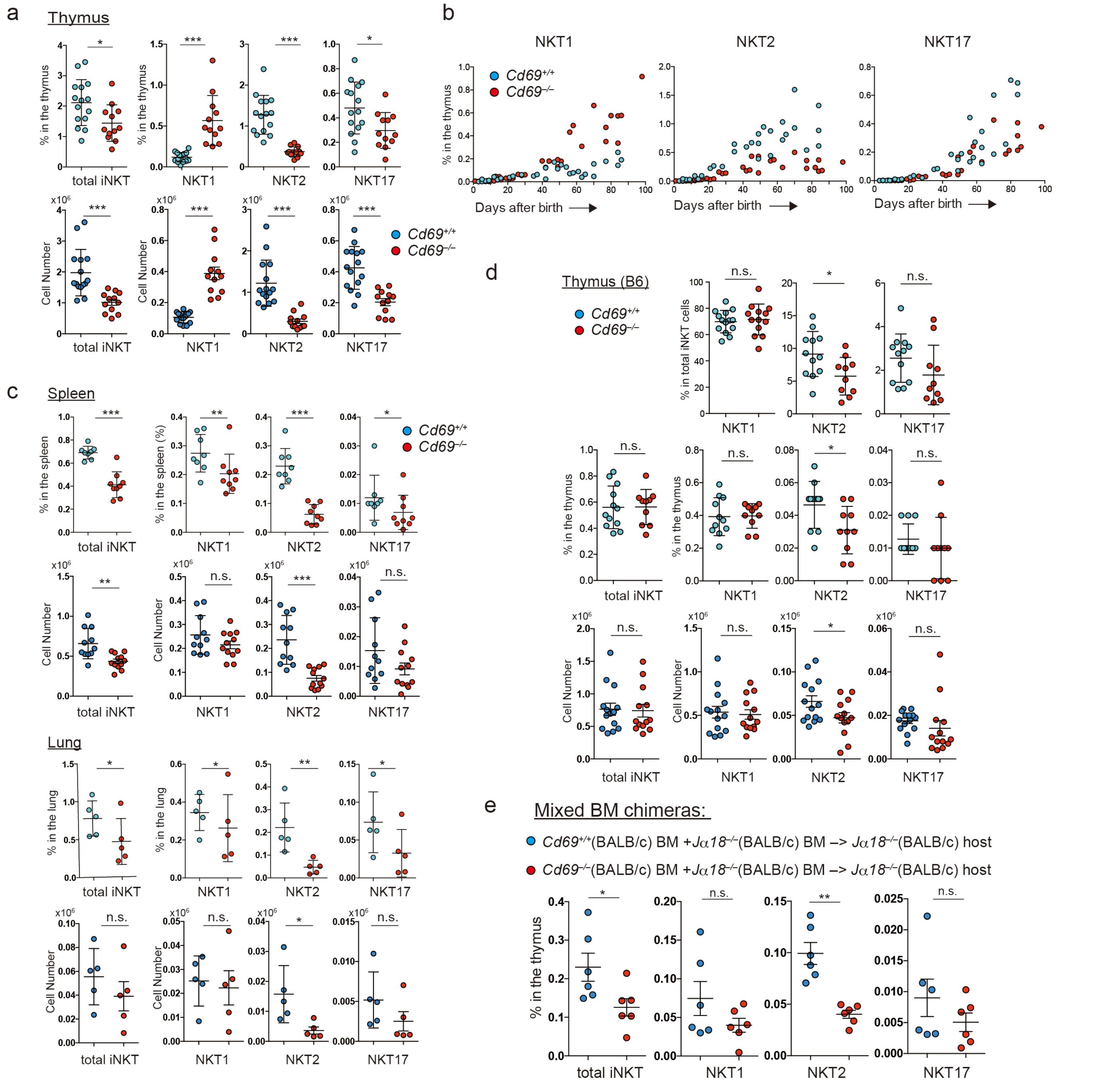

Supplementary Fig. 3: Diminished NKT2 cell development in *Cd69*$^{–/–}$ mice. (a) The total frequency of $CD3^+CD1d.PBS\text{-}57^+$ iNKT cells and the frequencies of NKT1 (PLZF^{lo}T-bet$^+$), NKT2 (PLZF^{hi}Rorγt^{med}) and NKT17 (PLZF^{med}Rorγt^{hi}) cells in the BALB/c thymus (upper) along with the absolute numbers of each population (bottom) are shown. (b) Ontogeny of iNKT subsets in the BALB/c thymus. The frequencies of NKT1, NKT2 and NKT17 cells (identified as in Supplementary Fig. 3a) in the thymus up to 100 days after birth are shown. (c) The total frequency of $CD3^+CD1d.PBS\text{-}57^+$ iNKT cells and the frequencies of NKT1, NKT2 and NKT17 cells (identified as in a) in the spleen and lung of BALB/c-background mice (upper) along with the absolute numbers of each population (bottom) are shown. (d) The total frequency of CD3 $^+$CD1d.PBS-57$^+$ iNKT cells and the frequencies of NKT1, NKT2 and NKT17 cells (identified as in a) in total iNKT cells (upper) and in the thymus (middle) from C57BL/6-background mice along with the absolute numbers of each population (bottom) are shown. (e) The frequency of total iNKT, NKT1, NKT2 and NKT17 cells in the thymus from the mixed BM chimeras in which *Cd69*$^{+/+}$ (BALB/c) or *Cd69*$^{–/–}$ (BALB/c) BM cells together with *Jα18*$^{–/–}$ (BALB/c) BM cells were transferred into lethally irradiated *Jα18*$^{–/–}$ (BALB/c) host mice. The mean and S.E.M. are shown. *p<0.05, **p<0.01, ***p<0.001 (two-tailed unpaired *t*-test). Representative data from 8-11 experiments with more than 10 mice are shown (a). Data are pooled from more that 10 experiments with 30 (*Cd69*$^{+/+}$) or 37 (*Cd69*$^{–/–}$) mice (b), 4-8 experiments with more than 8 mice (spleen) or 5 mice (lung) (c), 10 experiments with more than 10 mice (d) and 2 experiments with 6 mice (e).

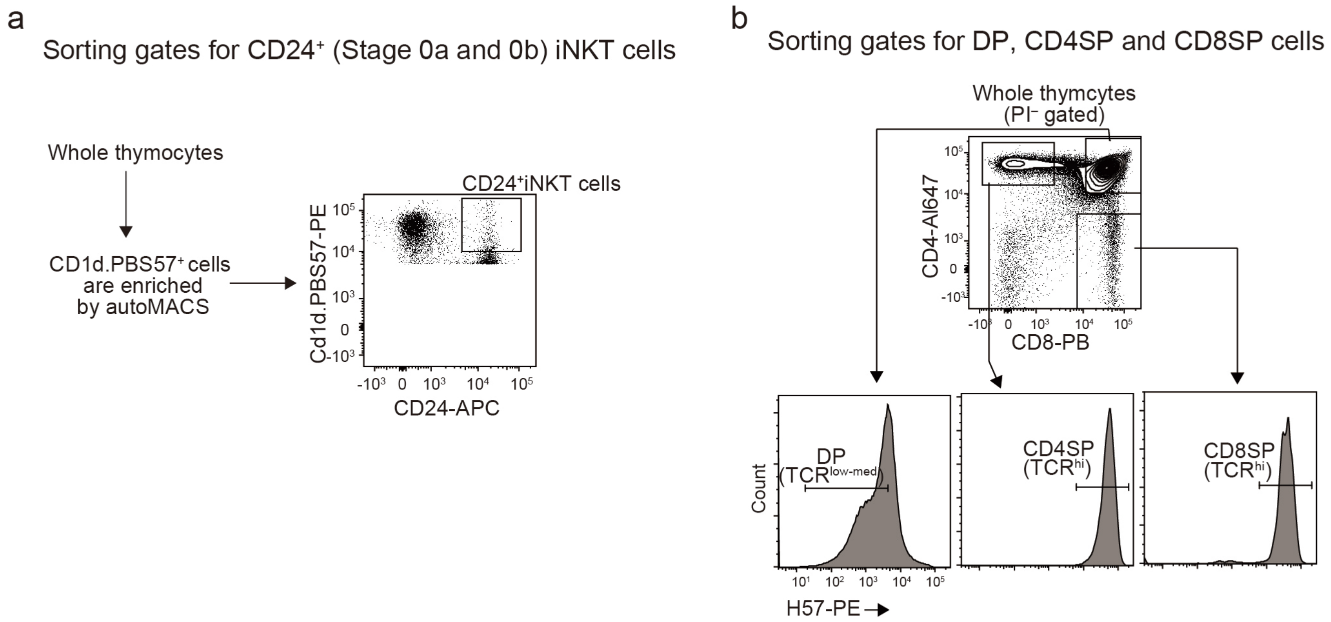

Supplementary Fig. 4: Gating strategies used for cell sorting. (a) Gating strategy to sort CD24$^+$ (stage 0a and 0b) iNKT cells from the thymus (Fig. 4a, 4f). (b) Gating strategy to sort DP (CD4$^+$CD8$^+$ TCR^{lo-med}), CD4SP (TCR^{hi}) and CD8SP (TCR^{hi}) cells from the thymus (Fig. 6e).

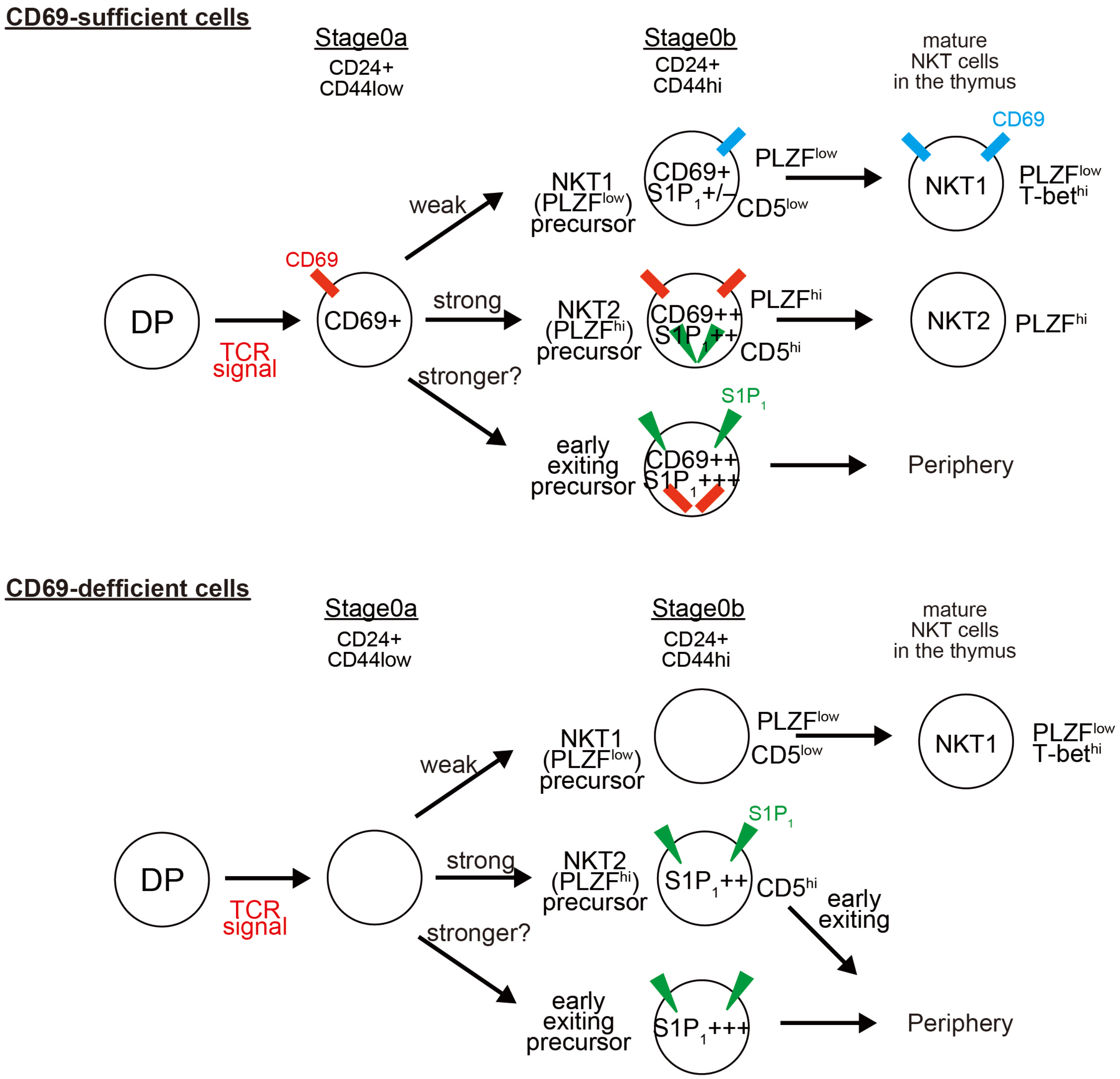

Supplementary Fig. 5: Model of iNKT cell development in the thymus. In CD69-sufficient cells, strong TCR signaling induces NKT2 (PLZF^{hi}) innate precursors with high CD69 and S1P$_1$ expression that become NKT2 cells, whereas weak TCR signaling induces NKT1 (PLZF^{low}) innate precursors with little S1P$_1$ and these cells become NKT1 cells. Some precursors that receive strong TCR signaling leave the thymus prematurely and are called ‘early-exiting precursors’. In contrast, CD69-deficient NKT2 (PLZF^{hi}) innate precursors express surface S1P$_1$ and prematurely leave the thymus, thus failing to become mature NKT2 cells. As a result, NKT1 cells become dominant in the thymus of *Cd69*$^{-/-}$ mice (bottom).

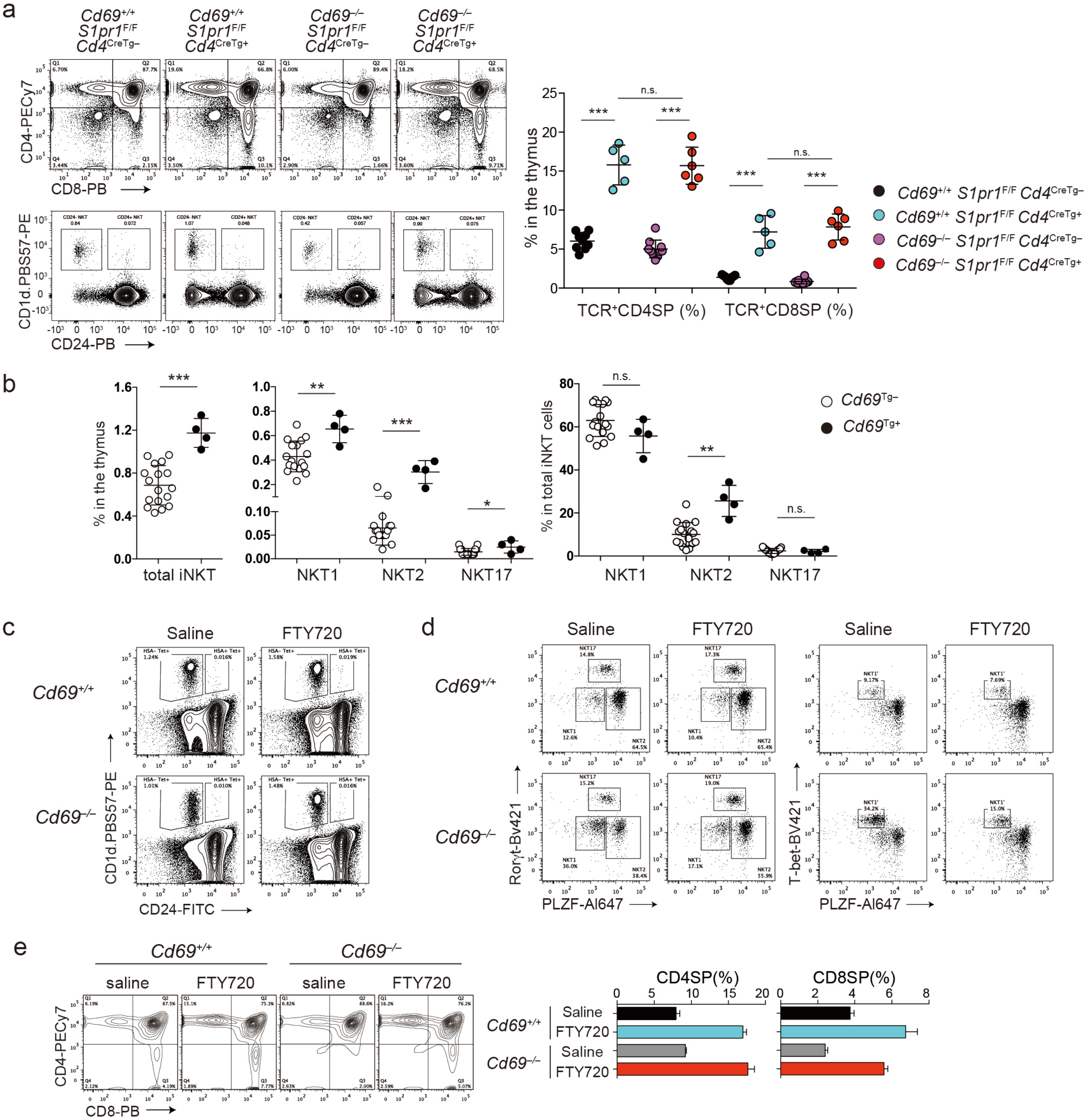

Supplementary Fig. 6: The influence of the prevention of S1P$_1$ expression on NKT2 cell generation. (a) The CD4/CD8 profiles (upper left) and CD24/CD1d.PBS57 profiles (bottom left) of the thymus from the indicated mice are shown. The frequencies of TCRβ$^+$CD4SP and TCRβ$^+$CD8SP cells are shown (right). (b) The total frequencies of CD3$^+$CD1d.PBS57$^+$ iNKT cells in the thymus from the indicated mice are shown (left). The frequencies of NKT1 (PLZF^{lo}T-bet$^+$), NKT2 (PLZF^{hi}Rorγt^{med}) and NKT17 (PLZF^{med}Rorγt^{hi}) cells in the thymus (middle) and among total iNKT cells (right) are shown. (c) The expression pattern of CD24 and CD1d.PBS57 in the thymus after five consecutive daily injections with either saline or FTY720 (S1P$_1$ agonist) is shown. (d) The expression patterns of PLZF and Rorγt or T-bet on gated CD3$^+$ CD1d.PBS57$^+$ iNKT cells are shown to identify NKT1, NKT2 and NKT17 cells. (e) The CD4/CD8 profiles of *Cd69*$^{+/+}$ and *Cd69*$^{-/-}$ thymi after five consecutive daily injections with either saline or FTY720. The frequencies of TCRβ$^+$CD4SP and TCRβ$^+$CD8SP cells in the thymus are shown. The mean and S.E.M. are shown. *p<0.05, **p<0.01, ***p<0.001 (two-tailed unpaired *t*-test). Representative data from 3-7 experiments with 5-10 mice (a) and 3 experiments with 3-5 mice (b, c, d, e) are shown.

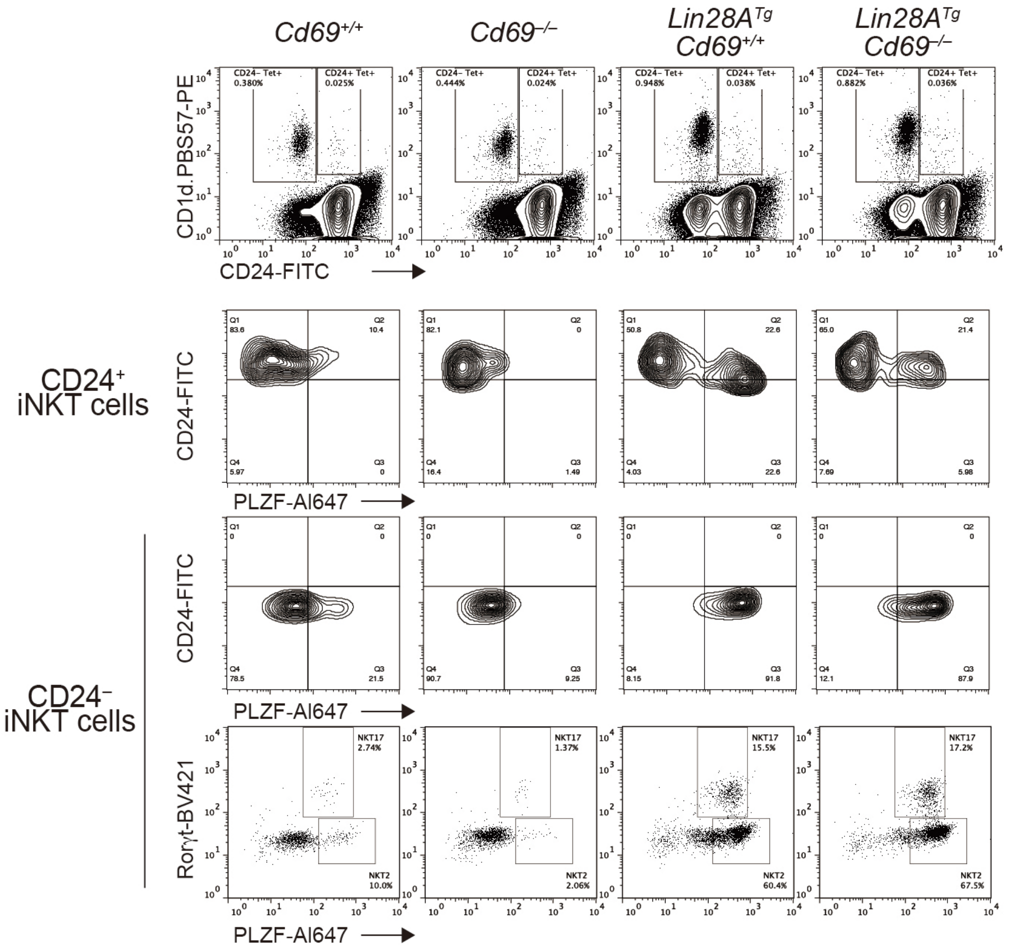

Supplementary Fig. 7: Profile of iNKT cells in the thymus of Lin28ATg. The expression pattern of CD24 and CD1d.PBS57 in the thymus is shown (upper). The PLZF expression on CD24^{+} iNKT cells (second row) and CD24^{-} iNKT cells (third row) is shown. The Rorγt and PLZF expression in gated CD24^{-}CD3^{+}CD1d.PBS57^{+} iNKT cells of the thymus from the indicated mice is shown (bottom). Representative data from two experiments with more than four mice (Lin28ATg) are shown.

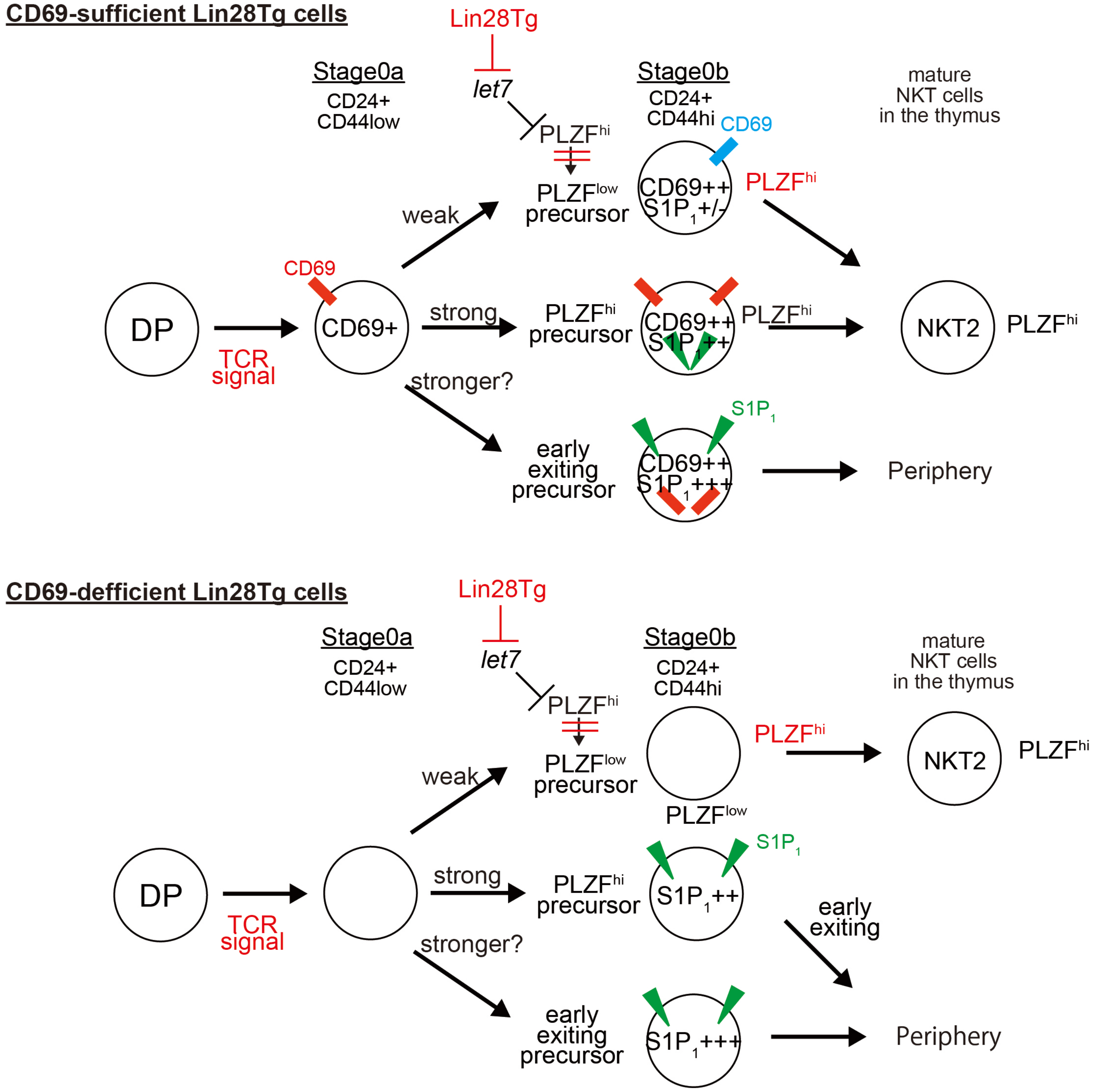

Supplementary Fig. 8: Model of iNKT cell development in the thymus of Lin28ATg mice. In CD69-sufficient cells, strong TCR signaling induces NKT2 (PLZF^{hi}) innate precursors; whereas weak TCR signaling induces NKT1 (PLZF^{low}) innate precursors which have high let-7 miRNA that prevents PLZF expression. In CD69-sufficient Lin28ATg mice, Lin28ATg inhibits the let-7 miRNA expression; therefore, the PLZF^{low} innate precursors induced by weak TCR signaling express high levels of PLZF. These cells then develop into NKT2 cells instead of NKT1 cells (top). In CD69-deficient Lin28ATg mice, the strongly TCR signaled NKT2 precursors leave the thymus prematurely and fail to develop into NKT2 cells. However, Lin28Tg prevents let-7 miRNA expression and thus leads to high PLZF expression in weakly signaled NKT1 precursors that then develop into NKT2 cells in the thymus (bottom).

Gene Name	Probe	Forward primer	Reverse primer
Rpl13a	108	ccctccaccctatgacaaga	gccccaggtaagcaaactt
Cd69	29	aacggaaaatagctcttcacatct	tgatgcttctcaaaatgtatactgg
Egr2	60	ctacccggtggaagacctc	aatgttgatcatgccatctcc
Eomes	51	caaagcggacaataacatgc	tggtgagttttaacttcccaaag
Il-4	92	cctgctcttctttctggaatgt	cacatccatctccgtgcat
S1pr1	66	cggtgtagacccagagtcct	agcttttccttggctggag
Klrb1c	69	ttggcatgagtcacccctat	gcttcagagccaacctgtgt
Klrb1b	2	ggcacagctttcaattctgat	tgtctgaagaacagccctca
Klrb1f	47	tgctcaagagaacaggactgaa	ggtctggaaatttgagaaacaaa
Klre1	26	gggaagaccattcggttctac	tgcacagaagtttttcatattagctt
Klrd1	88	ggattggaatgcattatagtgaaaa	tgctctggcctgataactgag
Klrk1	31	gatggctcctctctctcatacaa	tgagccatagacagcacagg
Klrc2	15	agtttggggtcctgcccttt	ccagtccatgagaccagtga
Klrc1	106	tctgaaacacctgcactgga	gaaagctgatgatcctttgga
Ly49A	38	ccagagaagctggctacagaa	tgccaacactgaaacagttacc
Rorc	6	acctcttttcacgggagga	tcccacatctcccacattg
Tbx21	106	agggggcttccaacaatg	agacgtgtgtgttagaagcactg
Zbtb16	88	agcctttgtgtgtgatcagtgt	gtttcccacacagcagacag

Supplementary Table 1: The primer sequences for quantitative RT-PCR
